# A Retrospective Analysis of Chemical Constituents in Regulated and Unregulated E-Cigarette Liquids

**DOI:** 10.3389/fchem.2021.752342

**Published:** 2021-10-28

**Authors:** Alaina K. Holt, Justin L. Poklis, Michelle R. Peace

**Affiliations:** ^1^ Department of Forensic Science, Virginia Commonwealth University, Richmond, VA, United States; ^2^ Integrative Life Sciences Doctoral Program, Virginia Commonwealth University, Richmond, VA, United States; ^3^ Department of Pharmacology and Toxicology, Virginia Commonwealth University, Richmond, VA, United States

**Keywords:** e-cig, olivetol, vitamin E, menthol, caffeine, ethanol, cannabinoids, vaping

## Abstract

E-cigarette or vaping use-associated lung injury (EVALI) was identified with the incidents of a multi-state outbreak of acute lung injuries associated with the use of electronic cigarettes (e-cigs) and attributed to vitamin E acetate in off-market cannabis-based e-liquids. Aside from EVALI, hypersecretion of mucus, irritated nasal passages, and watery, red eyes have been defined as complaints associated with vaping standard nicotine-based e-liquids. The chemical composition of e-liquids varies between manufacturers and robust oversight of ingredients is lacking. Manufacturers use chemicals deemed “generally recognized as safe” (GRAS) by the FDA, a designation for chemicals used in foodstuffs to be ingested. Most “GRAS” chemicals are associated with at least one Global Harmonization System (GHS) warning class, ranging from irritant to toxic. Untargeted chemical analysis is critical to evaluate e-liquid products to determine chemical composition; equally important is the quantitation of components to help elucidate the potential harms from exceeding recommended exposure limits. Untargeted screening of e-liquids was accomplished using gas chromatography-mass spectrometry (GC-MS) and Direct Analysis in Real Time-AccuTOF™ mass spectrometry (DART-ToF-MS) and has identified 350 chemical constituents from 241 products analyzed. Nicotine, caffeine, menthol, and vitamin E were confirmed and quantitated by GC-MS, ethanol was confirmed and quantitated by headspace-gas chromatography-dual flame ionization detection (HS-GC-FID), and olivetol and cannabinoids were confirmed and quantitated by liquid chromatography-tandem mass spectrometry (LC-MS/MS). Maximum identified concentrations of nicotine, caffeine, menthol, vitamin E, ethanol, olivetol, Δ9-tetrahydrocannabinol, and cannabidiol were 56.4, 26.9, 4.28, 307.9, 217.2, 399.6, 497.7, and 332.6 mg/ml, respectively. Evaluation of untargeted analysis and quantitation of unlabeled chemical components of e-liquids is essential to improving etiology of acute lung injury and less severe impacts of vaping, both short-term and long-term. The historical documentation of unlabeled ingredients can provide some insight for a retrospective analysis of health consequences and inform policy discussions.

## Introduction

The modern electronic cigarette evolved rapidly after its introduction in 2003 in the United States. Four device types are recognized by the Centers for Disease Control and Prevention (CDC). The first-generation device, the “cig-alike”, was low voltage and disposable and the second generation was re-fillable while the third generation enabled a user to select power, wicking material, and coil type. The fourth generation, called the “pod mod” allowed the user discreet vaping with small concealable devices and lack of associated aerosol cloud (Centers for Disease Control and Prevention, 2020a). Each type of e-cigarette allowed the consumer to make choices in line with their preferences. A fifth class of e-cigarette devices that facilitate the consumption of drugs other than nicotine (DOTN) and drug formulations that include waxes, dabs, crystals, and plant materials emerged from the highly customizable third generation device (Poklis et al., 2017a; Harrell and Eissenberg, 2018).

The liquid formulations used in the products, often referred to as e-liquids, also evolved as user preferences and public health sentiment changed, the industry evolved, and looming regulations became enforced. In addition to nicotine, typical e-liquid compositions contain humectants such as propylene glycol (PG) and vegetable glycerin (VG), an array of flavoring compounds, and other chemicals that are solvents for the flavorants or serve unknown purposes (Allen et al., 2016; Farsalinos et al., 2016; Peace et al., 2016; Poklis et al., 2017b; Peace et al., 2018; Fagan et al., 2018; Omaiye et al., 2019a; Holt et al., 2021). The cannabis industry drove an evolution of e-liquid formulations. Cannabinoids like Δ9-tetrahydrocannabinol (Δ9-THC) and cannabidiol (CBD) do not easily dissolve in PG and VG, which are hygroscopic. Medium chain triglycerides (MCT) and polyethylene glycol (PEG) have been used to dissolve the cannabinoids more easily in PG and VG or as stand-alone carriers (Troutt and DiDonato, 2017; Erickson, 2019; Muthumalage et al., 2020a).

Adverse effects from vaping nicotine-based e-liquids have been reported to include cough, airway irritation, mucus hypersecretion, red eyes, sinus irritation, cardiovascular damage, and pulmonary granulomas (Lestari et al., 2018; Thirion-Romero et al., 2019; Lin et al., 2020; Münzel et al., 2020; Overbeek et al., 2020).

In 2019 in the United States, e-cigarette or vaping use-associated lung injury (EVALI), a new type of lung injury directly related to e-cig use, emerged. As of February 18, 2020, 2,807 cases of EVALI hospitalizations, including 68 deaths, were reported by the CDC (Centers for Disease Control and Prevention, 2020b). Illicit cannabis products were most commonly reported by EVALI patients (82%), though some reported only using nicotine-based products (14%) (Ellington et al., 2020; Krishnasamy et al., 2020). In late-2019, the Minnesota Department of Health evaluated 46 THC-containing products submitted by 12 EVALI patients, identifying vitamin E acetate (VEA) in 52% of products, MCTs in 43%, CBD in 43%, and alpha-tocopherol (vitamin E, VE) in 37% (Taylor et al., 2019). Similar studies have also identified VEA in a high percentage of THC-containing products (Muthumalage et al., 2020a; Duffy et al., 2020). In 2020, the CDC concluded that VEA was a likely cause, as it was identified in most EVALI patients’ bronchoalveolar-lavage fluid along with a high percentage of products, but recognized that there may be more than one cause and that continued research is necessary (Blount et al., 2020; Ellington et al., 2020).

In May 2016, the FDA promulgated regulations to govern e-liquids, yet product approval deadlines were slated for May 2020, marking a significant 4 years delay in required compliance (United States Food and Drug Administration, 2020b). The flavoring ban instituted in January 2020 was an attempt to thwart adolescent usage, but only governed pod-based products (Yingst et al., 2021). While chemicals used to achieve particular flavor profiles were banned in pod-based formulations, chemicals used in e-liquid formulations were not dictated or restricted by the FDA’s regulatory language. The majority of flavorants used in e-liquids are substances which are Generally Recognized As Safe (GRAS) by the FDA. This designation is only applicable to food and food additives to be consumed orally (Flavor and Extracts Manufacturers Association of the United States, 2021). The presence of ethanol in e-liquids is documented, along with a number of other pharmacologically active chemicals (Peace et al., 2017; Poklis et al., 2017b; Poklis et al., 2017a; Poklis et al., 2019). Many e-liquids are marketed as containing vitamins and other health supplements, such as caffeine or melatonin. The addition of DOTNs less soluble in PG or VG required manufacturers to begin using other carriers, thus expanding the potential ingredient list consumer’s may be exposed to.

In addition to flavoring chemicals, e-liquids contain chemicals to achieve a desired consistency and pharmacological profile. Nicotine content in e-liquids increased with the introduction of nicotine salts, which are reported to make higher concentrations of nicotine more palatable to users (Harvanko et al., 2020). Other countries, like Canada and England, have regulations regarding allowable nicotine content in e-liquid formulation (Institute for Global Tobacco Control, 2018). To date, the FDA has not defined a maximum nicotine concentration. As vaping devices have evolved, so too have the options for using these devices to administer drugs other than nicotine (DOTN).

The following report highlights the findings from the untargeted evaluation of e-liquid products submitted or purchased for a comprehensive chemical analysis since 2014. Chemical profiles were generated using Direct Analysis in Real Time-AccuTOF™ mass spectrometry (DART-ToF-MS), gas chromatography mass spectrometry (GC-MS), head space gas chromatography with a flame ionization detector (HS GC-FID). Quantitation of targeted analytes was performed by GC-MS or liquid chromatograph tandem mass spectrometry (LC-MS/MS). The implications to health and safety of specific chemicals or classes of chemicals are also discussed.

## Materials

Since 2014, 241 e-liquids were submitted for analysis by individuals, purchased directly from manufacturers, or purchased from local retailers for product characterization.

All glassware, tubing, and fritted glass dispersion tubes were purchased from Colonial Scientific (Richmond, VA, United States). United States Pharmacopeia (USP) grade propylene glycol (PG) and vegetable glycerin (VG) were purchased from Wizard Labs (Altamonte Springs, FL, United States). HPLC grade acetone and Optima grade formic acid, isopropanol and methanol were purchased from Fisher Scientific (Hanover Park, IL, United States). 200-proof ethanol was purchased from Decon Labs (King of Prussia, PA, United States). T-butanol was purchased from Honeywell Riedel-de Haën (Seelze, Germany). Air, helium, hydrogen, and nitrogen gases were purchased from Praxair (Richmond, VA, United States) or AirGas (Richmond, VA, United States). Type 1 water was generated in-house using a Millipore Direct-Q3 system. (-)-Nicotine [≥99% (GC)], quinoline (reagent grade, 98%), caffeine, caffeine-(trimethyl-d9), menthol, and *trans*-anethole were purchased from Sigma (St. Louis, MO, United States). Certified reference materials for a quality assurance test mix containing amitriptyline, diazepam, fluoxetine, methadone, nicotine, nordiazepam, norfluoxetine, nortriptyline, paroxetine, and trazodone were acquired from Cerilliant (Round Rock, TX, United States), as were CBD, CBD-d3, Δ9-THC, Δ9-THC-d3, cannabigerol (CBG), cannabidivarin (CBDV), cannabichromene (CBC), cannabinol (CBN), CBN-d3, VE, VE-d6, and VEA. Tetrahydrocannabinolic acid (THCA), Δ8-tetrahydrocannabinol (Δ8-THC), cannabidiolic acid (CBDA), and cannabigerolic acid (CBGA) were purchased from Cayman Chemical (Ann Arbor, MI, United States). Olivetol was purchased from Santa Cruz Biotechnology (Santa Cruz, CA, United States) and olivetol-d9 was purchased from Toronto Research Chemicals (Toronto, ON, Canada).

## Methods

Lab protocol is to screen new products to elucidate chemical constituents and prioritize further analyses. Additional methods include confirmation and quantitation of nicotine and cannabinoids in all samples which had positive screen results. Volatiles and other chemicals of interest were also confirmed and quantitated. New quantitative methods were developed and validated as chemicals of interest were identified.

### Screening by DART-MS

Initial screening of e-liquids was performed on a JEOL JMS T100LC Accu-ToF DART-MS using a previously published method (Poklis et al., 2015). In brief, a capillary tube was dipped directly into the e-liquid and then introduced into the helium stream for analysis to identify components’ exact mass. The data was analyzed by creating an averaged, background subtracted, centroided mass spectra that was calibrated using PEG 600. Data was evaluated using National Institute of Standards and Technology (NIST) and Scientific Working Group for the Analysis of Seized Drugs (SWGDRUG) libraries. Full method details can be found in Supplementary Material A.

### Screening by GC-MS

E-liquids were also screened using a simple dilute-and-shoot preparation with an untargeted analysis method employed on a Shimadzu QP-2020 GC-MS (Kyoto, Japan). Samples were processed using NIST 17-1, 17-2, and 17s libraries and SWGDRUG 3.5-3.9 libraries for identification. Full method details can be found in Supplementary Material A.

### Quantitation of Nicotine by GC-MS

Quantitation of nicotine was accomplished by GC-MS using a Shimadzu QP-2020 GC-MS following previously published parameters (Pagano et al., 2016). Full method details can be found in Supplementary Material A.

### Quantitation of Caffeine and Menthol by GC-MS

Quantitation of caffeine and menthol was accomplished by developing a single-ion-monitoring method for GC-MS using a Shimadzu QP-2020 GC-MS. Full method details can be found in Supplementary Material A.

### Quantitation of Vitamin E and Vitamin E Acetate by GC-MS

Quantitation of VE and VEA was accomplished by developing a single-ion-monitoring method for GC-MS using a Shimadzu QP-2020 GC-MS. Full method details can be found in Supplementary Material A.

### Quantitation of Volatiles by HS-GC-FID

Quantitation of acetone, ethanol, isopropanol, and methanol was accomplished using a modified version of a previously published method for headspace gas chromatography-flame ionization detector (HS-GC-FID) (Poklis et al., 2017b) and employed a Shimadzu HS-20 headspace sampler attached to a Nexis 2030 GC-dual FID (Shimadzu Corp., Kyoto, Japan). Full method details can be found in Supplementary Material A.

### Quantitation of Olivetol by LC-MS/MS

Quantitation of olivetol was accomplished by developing a multiple-reaction-monitoring method for LC-MS/MS using a Shimadzu LC-MS 8050. Full method details can be found in Supplementary Material A.

### Quantitation of Cannabinoids by LC-MS/MS

Quantitation of cannabinoids was accomplished using a modified version of a previously published method with a Shimadzu LC-MS 8050 (Poklis et al., 2010). Full method details can be found in Supplementary Material A.

## Results

In all, 350 chemicals were identified among the 241 products evaluated (Supplementary Material C). Some products contained novel psychoactive substances (NPS), pharmaceuticals, or pharmacologically active herbal compounds. Table 1 highlights the major pharmacologically active ingredients identified through the screening process.

**TABLE 1:** Pharmalogically active chemicals identified in products through screening, with product type and frequency of detection.

Compound	Hazard class	Uses/Description	Product type								
			Nic (refill)	Nic (pod)	Non-nic (refill)	Nic/CBD (pod)	Cannab. (pod/cart)	Cannab. (refill)	Nic/DOTN (refill)	DOTN/non-nic (refill)	Additive
4-Fluoroisocathinone		Structurally mimics substituted cathinones and phenethylamines but avoids current legal issues by being neither							1	1	
4F-MDMB-BINACA		aka-4F-MDMB-BUTICANA or 4F-ADB; synthetic cannabinoid									1
5F-ADB	Irritant	aka-5F-MDMB-PINACA or 5F-ADB-PINACA; synthetic cannabinoid					1	2			1
5F-EDMB-PINACA		Synthetic cannabinoid									1
Apomorphine	Acute Toxic, Irritant, Health Hazard	Used as an alpha-adrenergic drug, a serotoneric drug, a dopamine agonist, and an emetic								1	
Caffeine	Irritant	CNS stimulant; anti-inflammatory and legal psychoactive that alters fatigue, mood, alertness, motor reaction time, vascular hemodynamics, and pain sensation		6							
Cannabidiol (CBD)	Acute Toxic, Irritant, Health Hazard	Active phytocannabinoid in Cannabis, but not psychoactive				1	11	11			2
Dextromethorphan (DXM)	Acute Toxic	Active ingredient in cough medicine; structural similarity to codeine and morphine									1
EMB-FUBINACA		aka - AEB-FUBINACA, FUB-AEB; synthetic cannabinoid									1
Ethanol	Flammable	Solvent/preservative; bactericidal activity/topical disinfectant; CNS depressant	32	2	7	1				5	
FUB-AMB	Irritant	aka - AMB-FUBINACA, MMB-FUBINACA; synthetic cannabinoid									1
γ-Butyrolactone (GBL)	Corrosive, Acute Toxic, Irritant	Flavorant; prodrug of Schedule 1 GHB; numerous legitimate industrial uses		3							
MDMB-FUBINACA		Synthetic cannabinoid								3	
MFUBINAC		Synthetic cannabinoid									1
Mitragynine	Irritant	A major component of kratom that acts via opioid receptors; stimulatory, antinociceptive, and opiate-like effects							2	2	
MMB-FUBICA		aka - AMB-FUBICA; synthetic cannabinoid									1
Nicotine	Acute Toxic, Environmental Hazard	Highly addictive CNS stimulant with many side effects; major component of cigarettes and often added to e-liquids	59	28		1					
Nuciferine	Acute Toxic	alkaloid of Blue Lotus								1	
Olivetol	Irritant	Precursor in various syntheses of tetrahydrocannabinol; people are claiming it can be used like Narcan for cannabis - helping to reduce a “raging high"				1	6				
Paynantheine		Major alkaloid found in kratom; thought to have cardiotoxic effects							1	1	
Tetrahydrocannabinol (d9-THC)	Irritant, Health Hazard	Schedule 1 drug; principal psychoactive compound in cannabis				1	8	3			
Tetrahydrocannabinolic acid (THCA)	Irritant, Health Hazard	Precursor of THC that converts through decarboxylation via heating					5	1			
Theobromine	Irritant, Health Hazard	Purine alkaloid derived from cacao and other plants. Is a vasodilator, diuretic, heart stimulator, bronchodilator, muscle stimulant. Similar to caffeine		6							

**FIGURE 1:**
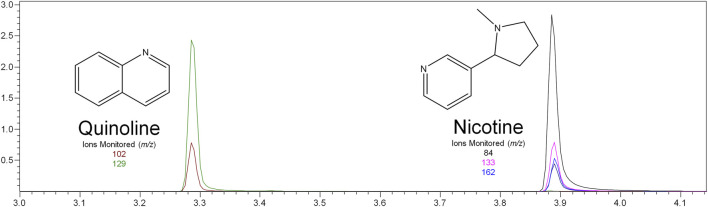
GC-MS chromatogram produced from nicotine standard (S6–500 μg/ml), with structures and ions monitored for nicotine and quinoline.

**FIGURE 2:**
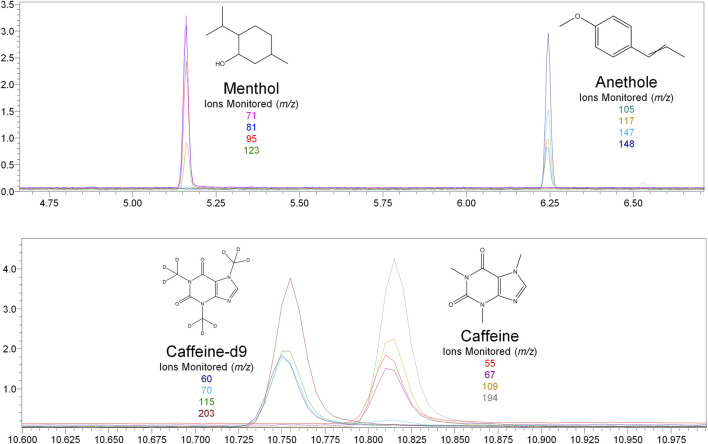
GC-MS chromatogram produced from mixed menthol and caffeine standard (S5–2000 ng/ml), with structures and ions monitored for menthol, anethole, caffeine, and caffeine-d9.

**FIGURE 3:**
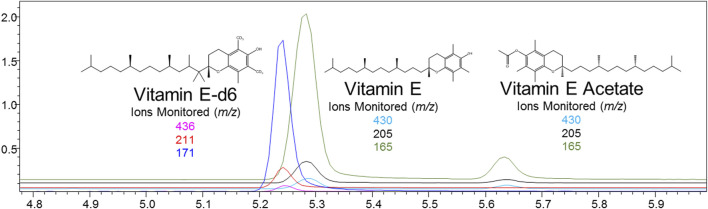
GC-MS chromatogram produced from mixed VE and VEA standard (S7–500 ng/ml), with structures and ions monitored for VE, VE-d6, and VEA.

**FIGURE 4:**
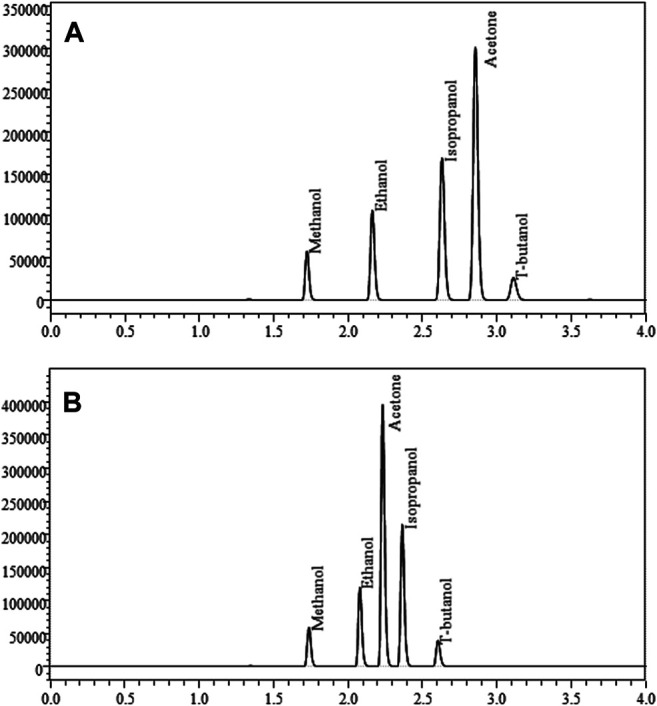
GC-FID chromatograms produced from quantitation of methanol, ethanol, isopropanol, and acetone using t-butanol as an internal standard. Figure A is a standard chromatogram produced by FID 1, and Figure B is a standard chromatogram produced by FID 2. Differences in analyte retention times are produced by the different separation techniques of the two chromatographic columns utilized.

**FIGURE 5:**
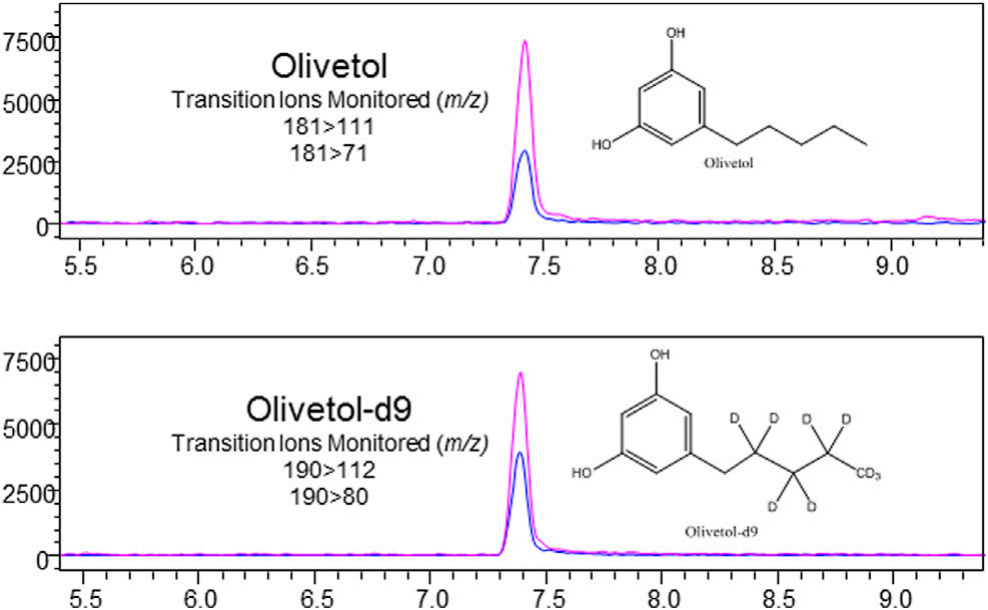
LC-MS/MS chromatogram produced from olivetol standard (S3–500 ng/ml), with structures and transitions monitored for olivetol and olivetol-d9.

**FIGURE 6:**
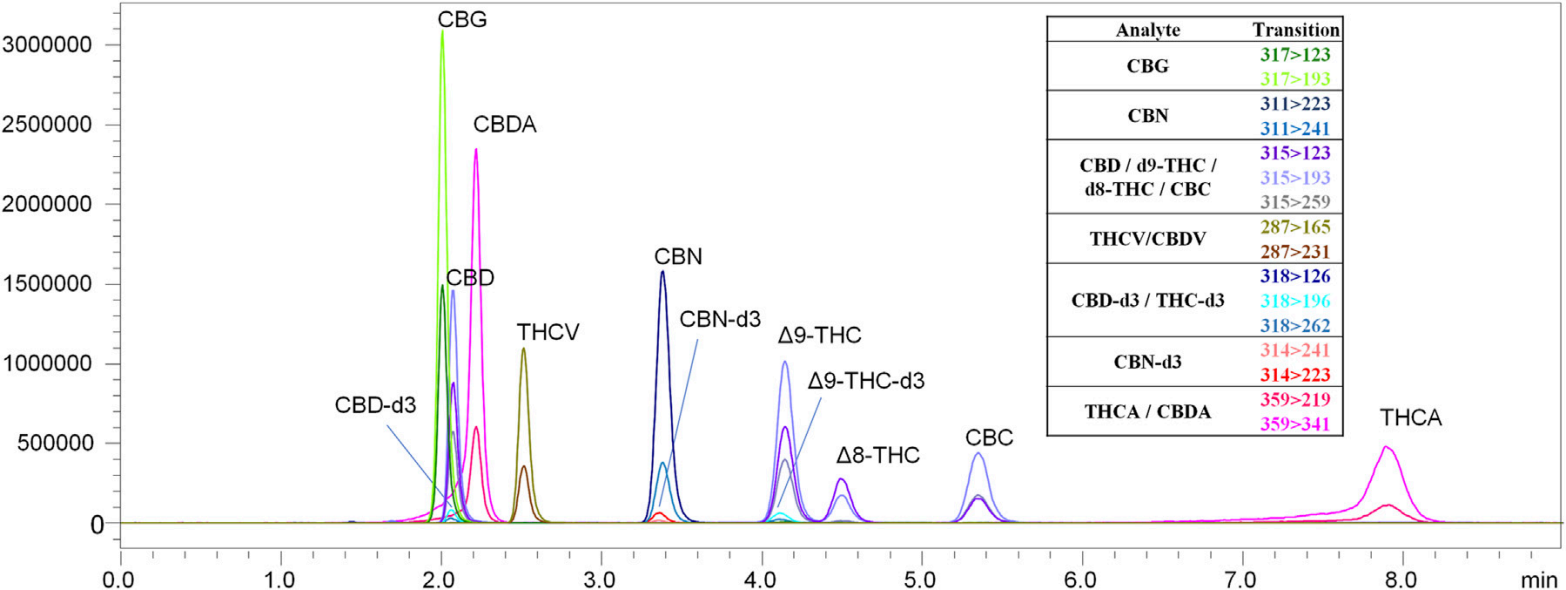
LC-MS/MS chromatogram produced from a mixed cannabinoid calibration standard. The chromatographic method employed was able to separate analytes with identical MRM transitions.

Nicotine has been quantitated in 90 products, with concentrations as high as 56.4 mg/ml. Figure 1 displays chromatography produced by the nicotine quantitation method, and individual product concentrations can be seen in Table 2. Nineteen products were evaluated for caffeine and menthol content, with quantitated concentrations as high as 26.9 µg/ml and 4.28 mg/ml, respectively. Figure 2 displays chromatography produced by the caffeine and menthol quantitation method, and individual product results can be seen in Table 3. Five products were evaluated for VE content, with quantitated concentrations as high as 307.9 μg/ml. VEA was not detected in any samples. Figure 3 displays chromatography produced by the VE and VEA quantitation method, and individual product concentrations can be seen in Table 4. 66 samples were evaluated for volatile content, in which ethanol has been quantitated in concentrations up to 217.2 mg/ml. Methanol and isopropanol were also identified but were below the limit of quantitation. Figure 4 displays chromatography produced by the volatiles quantitation method, and individual product results can be seen in Table 5. Olivetol has been quantitated for five samples, in concentrations as high as 399.6 μg/ml. Figure 5 displays chromatography produced by the olivetol quantitation method, and individual product results can be seen in Table 6. Cannabinoid content in the eleven products evaluated was found to vary greatly in both concentrations and compositions. CBD and Δ9-THC were the most identified cannabinoids and have been found in concentrations as high as 332.6 and 497.7 mg/ml, respectively. Cannabinoid concentrations identified through quantitation were often different from labeled values, as can be seen in Supplementary Material D. Figure 6 displays a chromatogram produced by the cannabinoid quantitation method. Individual cannabinoid transition ions monitored can be found in Supplementary Material B.

**TABLE 2:** Actual nicotine concentrations in e-liquids vs. labeled concentrations.

Brand	Product name	Labeled nicotine^a^ (mg/ml)	Actual nicotine^b^ (mg/ml)	% Difference	Product type
Adirondak	Delta	12.0	7.7	−43.7%	Nicotine e-liquid (refill)
AVAIL Vapor	Blueberry Cupcake	12.0 (1.2%)	12.0	0.0%	Nicotine e-liquid (refill)
AVAIL Vapor	Bombshell	6.0 (0.6%)	5.4	−10.5%	Nicotine e-liquid (refill)
AVAIL Vapor	Captain’s Cut	24.0 (2.4%)	24.0	0.0%	Nicotine e-liquid (refill)
AVAIL Vapor	Continental Breakfast	24.0 (2.4%)	24.0	0.0%	Nicotine e-liquid (refill)
AVAIL Vapor	Midnight Splash	24.0 (2.4%)	24.0	0.0%	Nicotine e-liquid (refill)
AVAIL Vapor	Persian Winter	24.0 (2.4%)	24.0	0.0%	Nicotine e-liquid (refill)
AVAIL Vapor	Sapphire Morning	18.0 (1.8%)	18.0	0.0%	Nicotine e-liquid (refill)
AVAIL Vapor	Wave Runner	3.0 (0.3%)	3.2	6.5%	Nicotine e-liquid (refill)
Bluumpod	Tobacco	50.0 (5%)	19.4	−88.2%	Nicotine/CBD e-liquid (pod)
Bombies	White Gummy Bear	6.0	5.0	−18.2%	Nicotine e-liquid (refill)
Bryce’s	Vanilla Cream Custard	6.0	5.9	−1.7%	Nicotine e-liquid (refill)
Coval Vapes	Mayflower	6.0	4.3	−33.0%	Nicotine e-liquid (refill)
Craft Sorbet	Skull Juice Watermelon Ice Cream	6.0	5.3	−12.4%	Nicotine e-liquid (refill)
Crafty E-Liquids	Watermelon	6.0	4.7	−24.3%	Nicotine e-liquid (refill)
Criss Cross	Original Tobacco	0.0	ND	—	Nicotine e-liquid (refill)
Elate Vapes	Hellcats	6.0	6.3	4.9%	Nicotine e-liquid (refill)
Fennet	High Janty	12.0	12.4	3.3%	Nicotine e-liquid (refill)
Five Pawns	Grandmaster	6.0	7.8	26.1%	Nicotine e-liquid (refill)
Glas	Menthol	50.0 (5%)	49.1	−1.8%	Nicotine e-liquid (pod)
Glas	Royal	50.0 (5%)	47.1	−6.0%	Nicotine e-liquid (pod)
Glas	Signature Tobacco	50.0 (5%)	40.7	−20.5%	Nicotine e-liquid (pod)
Good Life Vapor	El Kamino	12.0	8.7	−31.9%	Nicotine e-liquid (refill)
Gremlin Juice	Birthday Cake	12.0	11.6	−3.4%	Nicotine e-liquid (refill)
Gremlin Juice	Kentucky Mint Julep	6.0	6.3	4.9%	Nicotine e-liquid (refill)
Gremlin Juice	Vanilla Custard	12.0	9.1	−27.5%	Nicotine e-liquid (refill)
Hel Vapes	Nic Salt 50 mg	50.0	30.5	−48.4%	Nicotine e-liquid (refill)
Hel Vapes	Nic Salt 15 mg	15.0	8.8	−52.1%	Nicotine e-liquid (refill)
Hurricane	M B	18.0	4.4	−121.4%	Nicotine e-liquid (refill)
Hurricane	Watermelon	12.0	1.5	−155.6%	Nicotine e-liquid (refill)
Hurricane	Whiskey	12.0	3.3	−113.7%	Nicotine e-liquid (refill)
Indigo Vapor	Birthday Cake	12.0	10.8	−10.5%	Nicotine e-liquid (refill)
Indigo Vapor	Captain Ron	12.0	11.0	−8.7%	Nicotine e-liquid (refill)
Indigo Vapor	Sunset	6.0	6.0	0.0%	Nicotine e-liquid (refill)
Juice Head	Strawberry Kiwi	25	24.5	−2.0%	Nicotine e-liquid (refill)
Juice Mafia	Peach Tobacco	12.0	8.9	−29.7%	Nicotine e-liquid (refill)
Juice Mafia	Turkish Tobacco	12.0	11.2	−6.9%	Nicotine e-liquid (refill)
JUUL	Classic Tobacco 5% Nicotine	59.0	54.7	−7.6%	Nicotine e-liquid (pod)
JUUL	Classic Tobacco 3% Nicotine	35.0	32.3 [30.4–34.2]	−8.0%	Nicotine e-liquid (pod)
JUUL	Menthol 5% Nicotine	59.0	50.6	−15.3%	Nicotine e-liquid (pod)
JUUL	Menthol 3% Nicotine	35.0	34.3 [33.1–35.5]	−2.0%	Nicotine e-liquid (pod)
JUUL	Virginia Tobacco 5% Nicotine	59.0	56.4 [50.4–51.1]	−4.5%	Nicotine e-liquid (pod)
JUUL	Virginia Tobacco 3% Nicotine	35.0	31.6	−10.2%	Nicotine e-liquid (pod)
Mighty Vapors Salts	Hulk Tears	50	47.0	−6.2%	Nicotine e-liquid (refill)
Mt. Baker	GWAR Spew	12.0	13.3	10.3%	Nicotine e-liquid (refill)
My Ohms	Pink Melon	12.0	8.4	−35.3%	Nicotine e-liquid (refill)
NJOY	Blueberry	50.0 (5%)	46.1 [39.9–49.8]	−8.1%	Nicotine e-liquid (pod)
NJOY	Classic Tobacco	50.0 (5%)	44.7	−11.2%	Nicotine e-liquid (pod)
NJOY	Menthol	50.0 (5%)	46.2	−7.9%	Nicotine e-liquid (pod)
NJOY	Watermelon Twist	50.0 (5%)	47.6 [47.1–48.4]	−4.9%	Nicotine e-liquid (pod)
OG CBD Oil	Cookies ‘N Cream	0.0	ND	—	CBD e-liquid (pod)
OG CBD Oil	Mango	0.0	ND	—	CBD e-liquid (pod)
OG CBD Oil	Pineapple Express	0.0	ND	—	CBD e-liquid (pod)
OG CBD Oil	Pink Lemonade	0.0	ND	—	CBD e-liquid (pod)
Ritchy Group	Liqua Vanilla	0.0	<LOQ	—	Nicotine e-liquid (refill)
S&S Mods	Grumpy’s Hooch	12.0	9.0	−28.6%	Nicotine e-liquid (refill)
Seduce Juice	Jango	12.0	12.6	4.9%	Nicotine e-liquid (refill)
Seduce Juice	Pharoah	12.0	10.7	−11.5%	Nicotine e-liquid (refill)
Seduce Juice	Snake Eyes	12.0	10.1	−17.2%	Nicotine e-liquid (refill)
Seduce Juice	Snake Oil	12.0	10.5	−13.3%	Nicotine e-liquid (refill)
Shosha	Mango	12.0	12.0	0.0%	Nicotine e-liquid (refill)
Shosha	Seedless	18.0	16.3	−9.9%	Nicotine e-liquid (refill)
Shosha	USA Mix	12.0	13.4	11.0%	Nicotine e-liquid (refill)
Shosha	VG UAS	16.0	15.2	−5.1%	Nicotine e-liquid (refill)
Shosha	VG Virginia	16.0	15.2	−5.1%	Nicotine e-liquid (refill)
Sky Pod	Blue Lemonade	60 (6%)	33.2	−57.5%	Nicotine e-liquid (pod)
StL Vapor	Spearmint	22.0	10.0	−75.0%	Nicotine e-liquid (refill)
Supreme Nicotine	258 Rally Squirrel	16.0	10.3	−43.3%	Nicotine e-liquid (refill)
Top Vapor	Unflavored PG	6.0	8.3	32.2%	Nicotine e-liquid (refill)
Triumph	Juicy Peach	11.0	8.4	−26.8%	Nicotine e-liquid (refill)
VapeWell	Cheery	18.0	14.7	−20.2%	Nicotine e-liquid (refill)
Vapourium NZ	Caffiend	12.0	10.8	−10.5%	Nicotine e-liquid (refill)
Vapourium NZ	Deez Melons	12.0	11.1	−7.8%	Nicotine e-liquid (refill)
Vapourium NZ	Jamaican Rum	6.0	5.2	−14.3%	Nicotine e-liquid (refill)
Vapourium NZ	So Fresh So Clean	12.0	12.4	3.3%	Nicotine e-liquid (refill)
Vapourium NZ	Stoned Fruits	12.0	11.4	−5.1%	Nicotine e-liquid (refill)
Vapourium NZ	Vanilla Beanie	12.0	10.4	−14.3%	Nicotine e-liquid (refill)
Velvet Cloud Vapor	Vanilla Tobacco	6.0	6.5	8.0%	Nicotine e-liquid (refill)
Wizard Labs	VG	12.0	10.3	−15.2%	Nicotine e-liquid (refill)

a Nicotine concentrations advertised as a (percentage) have been converted to mg/ml for comparison.

b Average of multiple samples/lots, with [range].

**TABLE 3:** Caffeine and menthol concentrations in e-liquids.

Product^a^	Caffeine (µg/ml)	Menthol (µg/ml)	Product type
Appalachian Sunshine	ND	139.4 ± 6.1	CBD vape cart
Bluumpod “Tobacco”	ND	ND	Nicotine/CBD e-liquid
Glas “Fresh Menthol”	ND	4,278.6 ± 109.9	Nicotine e-liquid (pod)
Juul “Classic Tobacco” 3%	26.9 ± 1.3	49.9 ± 0.9	Nicotine e-liquid (pod)
Juul “Classic Tobacco” 3%	24.7 ± 0.6	96.2 ± 8.3	Nicotine e-liquid (pod)
Juul “Classic Tobacco” 5%	25.3 ± 0.1	118.4 ± 2.9	Nicotine e-liquid (pod)
Juul “Menthol” 3%	10.7 ± 0.5	1,361.8 ± 52.1	Nicotine e-liquid (pod)
Juul “Menthol” 3%	9.7 ± 0.2	1995.4 ± 18.6	Nicotine e-liquid (pod)
Juul “Menthol” 5%	8.8 ± 0.3	1,391.7 ± 31.4	Nicotine e-liquid (pod)
Liberty	ND	3.8 ± 0.4	CBD vape cart
Mighty Vapors Salts “Hulk Tears”	ND	ND	Nicotine e-liquid
Myle Mini-unknown flavor	ND	2,208.3 ± 126.3	Nicotine disposable
NJOY “Blueberry”	ND	182.1 ± 4.8	Nicotine e-liquid (pod)
NJOY “Blueberry”	ND	117.5 ± 3.73	Nicotine e-liquid (pod)
NJOY “Menthol”	ND	1750.2 ± 32.9	Nicotine e-liquid (pod)
NJOY “Watermelon Twist”	ND	3.2 ± 0.3	Nicotine e-liquid (pod)
NJOY “Watermelon Twist”	ND	10.8 ± 0.1	Nicotine e-liquid (pod)
Twisted CBD “Watermelon”	ND	24.4 ± 1.0	CBD vape cart
Vuse “Mixed Berry” 5%	ND	118.0 ± 1.3	Nicotine e-liquid (pod)

a Duplicate products from separate submissions or product lots.

**TABLE 4:** Vitamin E concentrations in e-liquids.

Product	Concentration (µg/ml)	Product type
Appalachian Sunshine	236.7 ± 7.5	CBD vape cart
Diamond CBD	2.9 ± 0.1	Dietary supplement/vape additive
Liberty	226.6 ± 23.1	CBD vape cart
MMS Elemental “Blue Dream”	307.9 ± 11.0	CBD vape cart
Unidentified THC product	101.6 ± 0.5	THC vape cart

**TABLE 5:** Ethanol concentrations in e-liquids.

Product	Average concentration (mg/ml)				Product type
	Methanol	Ethanol	Isopropanol	Acetone	
Aqua “Flow”	ND	1.7 ± 0.4	ND	ND	Nicotine e-liquid (refill)
Blue CBD “Crystals Isolate” 1,000 mg	ND	ND	ND	ND	CBD Additive
Blue CBD “Crystals Isolate” 250 mg	ND	ND	ND	ND	CBD Additive
Blue Monkey Vapes “Dr. Freeze-Ice Menthol”	ND	2.7 ± 0.1	ND	ND	Nicotine e-liquid (refill)
Bluumpod “Tobacco”	ND	<LOQ	ND	ND	Nicotine/CBD e-liquid
Cereal Killa “Duchess”	ND	9.6 ± 0.7	ND	ND	Nicotine e-liquid (refill)
Clown “Pennywise Circus Salts”	ND	13.1 ± 0.1	ND	ND	Nicotine e-liquid (refill)
Craft Sorbet Skull Juice “Watermelon Ice Cream”	<LOQ	<LOQ	ND	ND	Nicotine e-liquid (refill)
Crafty “Watermelon”	ND	5.5 ± 0.3	ND	ND	Nicotine e-liquid (refill)
Diamond CBD “Hemp Infused Liquid” 1,000 mg	ND	ND	ND	ND	CBD Additive
Diamond CBD “Hemp Infused Liquid” 50 mg	ND	ND	ND	ND	CBD Additive
Diamond CBD Vape Additive	ND	ND	ND	ND	CBD Additive
Directors Cut “The Devil”	ND	4.7 ± 0.1	ND	ND	Nicotine e-liquid (refill)
Elate Vape “Hell-Cats”	ND	ND	ND	ND	Nicotine e-liquid (refill)
Fresh Pressed “Fruit Finale”	ND	<LOQ	ND	ND	Nicotine e-liquid (refill)
Galaxy	ND	ND	ND	ND	Vape additive
Geeked Out “Dork Breath”	ND	3.7 ± 0.2	ND	ND	Nicotine e-liquid (refill)
HEL Vape “Breaking Bad” 15 mg Nic salt	ND	ND	ND	ND	Nicotine e-liquid (refill)
HEL Vape “Breaking Bad” 50 mg Nic salt	ND	ND	ND	ND	Nicotine e-liquid (refill)
Hurricane “M B”	ND	ND	ND	ND	Nicotine e-liquid (refill)
Hurricane “Watermelon”	ND	ND	ND	ND	Nicotine e-liquid (refill)
Hurricane “Whiskey”	ND	ND	ND	ND	Nicotine e-liquid (refill)
Jango	ND	ND	ND	ND	Nicotine e-liquid (refill)
JUUL Menthol	ND	2.0 ± 0.04	ND	ND	Nicotine e-liquid (pod)
JUUL Virginia Tobacco	ND	32.8 ± 0.7	ND	ND	Nicotine e-liquid (pod)
Kai’s Virgin Vapor “Blue Vango” 0 mg Nic	ND	2.6 ± 0.03	ND	ND	Non-nicotine e-liquid (refill)
Kai’s Virgin Vapor “Blue Vango” 12 mg Nic	ND	2.3 ± 0.1	ND	ND	Nicotine e-liquid (refill)
Kai’s Virgin Vapor “Caramel Kona Milkshake” 0 mg Nic	ND	89.5 ± 3.2	ND	ND	Non-nicotine e-liquid (refill)
Kai’s Virgin Vapor “Caramel Kona Milkshake” 12 mg Nic	ND	37.0 ± 1.4	ND	ND	Nicotine e-liquid (refill)
Kai’s Virgin Vapor “Celestial Honeydew” 0 mg Nic	ND	16.1 ± 0.7	ND	ND	Non-nicotine e-liquid (refill)
Kai’s Virgin Vapor “Celestial Honeydew” 12 mg Nic	ND	16.5 ± 0.6	ND	ND	Nicotine e-liquid (refill)
Kai’s Virgin Vapor “Chocolate Grasshopper” 0 mg Nic	ND	46.5 ± 1.4	ND	ND	Non-nicotine e-liquid (refill)
Kai’s Virgin Vapor “Chocolate Grasshopper” 12 mg Nic	ND	19.6 ± 1.9	ND	ND	Nicotine e-liquid (refill)
Kai’s Virgin Vapor “French Vanilla Kiss” 0 mg Nic	ND	214.3 ± 13.6	ND	ND	Non-nicotine e-liquid (refill)
Kai’s Virgin Vapor “French Vanilla Kiss” 12 mg Nic	ND	65.7 ± 3.4	ND	ND	Nicotine e-liquid (refill)
Kai’s Virgin Vapor “Plum Crazy” 0 mg Nic	ND	2.5 ± 0.04	ND	ND	Non-nicotine e-liquid (refill)
Kai’s Virgin Vapor “Plum Crazy” 12 mg Nic	ND	3.4 ± 0.2	ND	ND	Nicotine e-liquid (refill)
Kai’s Virgin Vapor “Raspberry Mocha Whip” 0 mg Nic	ND	39.2 ± 4.3	ND	ND	Non-nicotine e-liquid (refill)
Kentucky Route “Strawberry Fields”	ND	ND	ND	ND	Vape additive
Koi “Blue Raspberry Dragon Fruit”	ND	<LOQ	ND	ND	Vape additive
Lotus Extracts “Areca Nut”	ND	ND	ND	ND	DOTN e-liquid
Lotus Extracts “Blue Lotus”	ND	1.9 ± 0.05	ND	ND	DOTN e-liquid
Lotus Extracts “Damiana”	ND	ND	ND	ND	DOTN e-liquid
Lotus Extracts “Klip Dagga”	ND	<LOQ	ND	ND	DOTN e-liquid
Lotus Extracts “Kra Thum Kok”	ND	<LOQ	ND	ND	DOTN e-liquid
Lotus Extracts “Kra Thum Na”	ND	2.3 ± 0.1	<LOQ	ND	DOTN e-liquid
Lotus Extracts “Wild Lettuce”	ND	ND	ND	ND	DOTN e-liquid
My Ohm “Pink Melon”	ND	<LOQ	ND	ND	Nicotine e-liquid (refill)
R.A. Royal CBD “Classic”	ND	ND	ND	ND	Vape additive
Ritchy “Liqua Vanilla”	ND	<LOQ	ND	ND	Nicotine e-liquid (refill)
Shosha “Mango”	ND	<LOQ	ND	ND	Nicotine e-liquid (refill)
Shosha “Seedless”	ND	8.8 ± 0.5	ND	ND	Nicotine e-liquid (refill)
Shosha “UAS Mix”	ND	1.5 ± 0.02	ND	ND	Nicotine e-liquid (refill)
Shosha “USA Mix”	ND	1.6 ± 0.07	ND	ND	Nicotine e-liquid (refill)
Shosha “Virginia”	<LOQ	1.8 ± 0.07	ND	ND	Nicotine e-liquid (refill)
Triumph “Juicy Peach”	ND	ND	ND	ND	Nicotine e-liquid (refill)
Vapourium “Caffiend”	ND	<LOQ	ND	ND	Nicotine e-liquid (refill)
Vapourium “Deez Melons”	ND	<LOQ	ND	ND	Nicotine e-liquid (refill)
Vapourium “Jamaican Rum”	ND	<LOQ	ND	ND	Nicotine e-liquid (refill)
Vapourium “So Fresh So Clean”	ND	28.7 ± 0.7	ND	ND	Nicotine e-liquid (refill)
Vapourium “Stoned Fruits”	ND	17.0 ± 0.9	ND	ND	Nicotine e-liquid (refill)
Vapourium “Vanilla Beanie”	ND	<LOQ	ND	ND	Nicotine e-liquid (refill)
Whispers “Razzel Dazzel”	ND	<LOQ	ND	ND	Nicotine e-liquid (refill)
Yami Vapor “Joy Trio” 35 mg Nic	ND	2.3 ± 0.1	ND	ND	Nicotine e-liquid (refill)
Yami Vapor “Joy Trio” 50 mg Nic	ND	2.5 ± 0.1	ND	ND	Nicotine e-liquid (refill)
Yami Vapor “Taruto”	ND	16.5 ± 1.4	ND	ND	Nicotine e-liquid (refill)

**TABLE 6:** Olivetol concentrations in e-liquids.

Product	Concentration (µg/ml)	Product type
Appalachian Sunshine	3,880.9 ± 185.7	CBD vape cart
Bluumpod CBD “Tobacco”	<LOQ	Nicotine/CBD e-liquid
Liberty	8.6 ± 0.4	CBD vape cart
Twisted CBD “Watermelon”	21.4 ± 3.0	CBD vape cart
Western Cultured “Seatown Lemon Haze”	<LOQ	CBD vape cart

## Discussion

**FIGURE 7:**
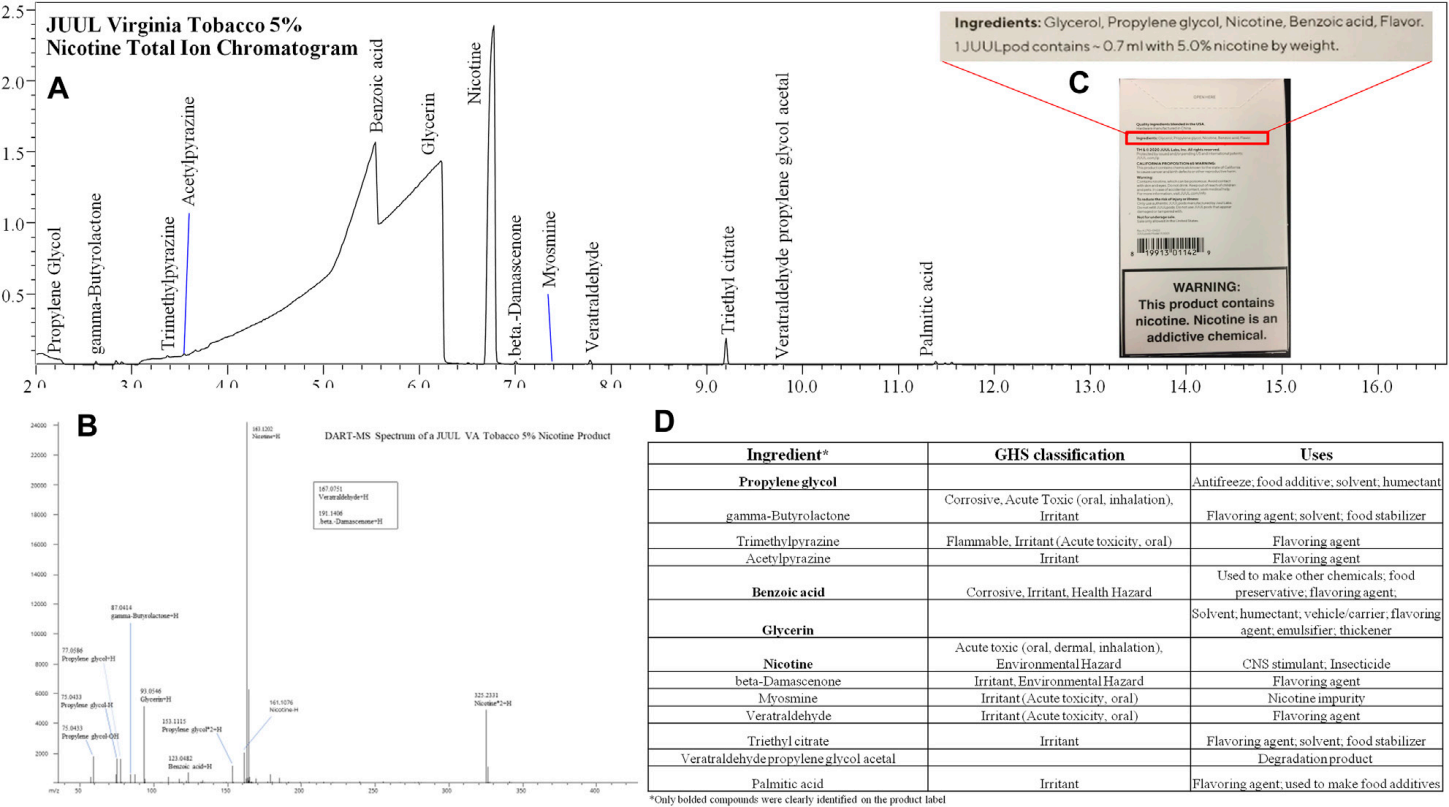
**(A)** JUUL Virginia Tobacco 5% Nicotine Total Ion Chromatogram, **(B)** DART-MS Spectrum, **(C)** Packaging, and **(D)** GHS classification and Uses of Identified Ingredients. This exemplifies the typical results of product screening.

All e-liquids were analyzed by DART-MS and GC-MS with an untargeted method to characterize the chemical profile. An example of screening results can be seen in Figure 7. Once the chemicals were identified, quantitative analysis was performed by HS GC-FID, GC-MS, or LC-MS/MS. The chemicals identified in this study can be classified as carriers or humectants, flavorants/organoleptics, preservatives, additives/enhancers, or as pharmacologically active. Several chemicals, such as menthol, have multiple properties, making attribution of their use in the e-liquid difficult. Supplementary Material C lists compounds identified, associated GHS hazard classes, and reported uses of compounds. Untargeted analytical methods are necessary to evaluate products for compliance with regulations and to assess unregulated products. With widely varied and constantly changing products like e-liquids, a critical evaluation of chemical composition is crucial for public health and safety. Although not reported here, analysis of the aerosols can identify if compounds in the e-liquid will be inhaled by the user. The aerosol analysis can also determine if new, unique compounds are formed from the vaping process, such as pyrolytic, degradant, or adduct compounds. Chemicals identified in e-liquids can indicate user exposure, though in the absence of aerosol studies, results should be interpreted with caution.

Of products evaluated in this study, the more specifically regulated nicotine e-liquids found in pod style products have trended toward more simple compositions. Nicotine based refill formulations, potentially associated with small-batch craft products, have continued to have complex chemical profiles, often containing more flavoring chemicals. For example, 10 chemicals were identified in a JUUL Menthol pod, whereas 26 chemicals were identified in Mighty Vapors “Hulk Tears”, a refill formulation. Cannabinoid-containing products also tend to have more chemical constituents, as many of the terpenes from cannabis are extracted along with the desired cannabinoids. For example, MMS Elemental “Blue Dream” contained 35 identified chemicals, 23 of which were terpenes, and 9 of which were cannabinoids. These terpenes can naturally add to the flavor and aroma profiles, but may also be added to formulations to simulate desired profiles. As with other compounds, these terpenes can have multiple properties, including GHS health hazards. The lack of regulations governing product ingredients provides manufacturers opportunity to create complicated products with potentially harmful chemicals.

Most carriers and flavorants identified in products are “generally recognized as safe” by the FDA, meaning “the substance is generally recognized, among qualified experts, as having been adequately shown to be safe under the conditions of its intended use, or unless the use of the substance is otherwise excepted from the definition of a food additive” (United States Food and Drug Administration, 2019; Flavor and Extracts Manufacturers Association of the United States, 2021). GRAS status only applies to foodstuffs to be ingested orally and does not translate to any other route of administration. Extrapolation of oral ingestion safety to inhalation safety is fraught with assumptions and false equivalencies.

A variety of chemical compounds with pharmacologically active properties were found in addition to nicotine, as seen in Table 1. This study identified cannabinoids (CBD, Δ9-THC, Δ8-THC, CBG, CBDA, CBN, CBC, THCV, and THCA), caffeine, dextromethorphan, kratom alkaloids (mitragynine and 7-hydroxy-mitragynine), the blue lotus alkaloids apomorphine and nuciferine, gamma-Butyrolactone (GBL), a variety of synthetic cannabinoids (5F-ADB, MDMB-FUBINACA, FUB-AMB, EMB-FUBINACA, MFUBINAC, MMB-FUBICA, 4F-MDMB-BINACA, 5F-EDMB-PINACA) and the synthetic cathinone 4-fluoroisocathinone in products. Nicotine, Δ9-THC, and CBD were the only active ingredients identified on the product labels. All other pharmacologically active ingredients were unlisted on the products, meaning consumers did not know what they were consuming. Some of the pharmacologically active ingredients are illicit substances, some are legal herbal compounds, and others are co-opted therapeutic drugs used for recreational purposes.

### Carriers, Diluents, and Thickeners

Propylene glycol and vegetable glycerin were the most commonly identified carriers in e-liquids. Both are considered GRAS and used in food products, pharmaceuticals, and health and beauty products. Though considered harmless based on years of use in food and medicine, use of these chemicals in e-cigs and vaporizers is not inherently safe. Studies have demonstrated that when heated to high temperatures, like those of a heated e-cig coil, PG and VG can produce carbonyls such as formaldehyde and acetaldehyde (Kosmider et al., 2014; Geiss et al., 2016; Troutt and DiDonato, 2017; Qu et al., 2019; Jiang et al., 2020). Device settings have been found to directly affect the production of these harmful chemicals, and therefore may lead to the risks of increased exposures to these carcinogens, as carbonyl formation has been shown to increase directly with increasing battery output voltages (Kosmider et al., 2014; Qu et al., 2019).

PG and VG were the only known carriers identified in nicotine and flavorant-only formulations. In addition to PG and VG, MCTs, PEG, and squalene were identified in cannabinoid and DOTN formulations, as well as formulations that contained both nicotine and a DOTN. These carriers may be used to dissolve more lipophilic compounds, including cannabinoids, before adding them to PG and VG. MCTs may also be used because they are attributed with health benefits. MCTs have been touted online as a safer, healthier alternative to PG and VG (Zachar, 2018). MCTs produce harmful volatile organic compounds and increase interleukin-8 (IL-8) and interleukin-6 (IL-6) levels which are biomarkers for lung inflammation and injury. MCTs also decrease transepithelial electrical resistance and increase lipid-laden macrophage formation which can lead to lipoid pneumonia (Muthumalage et al., 2020b). MCT aerosols were found to contain alkyl alcohols, which are surfactant-like and can produce cytotoxic effects. MCTs and PEG both produce harmful carbonyls when aerosolized (Troutt and DiDonato, 2017; Jiang et al., 2020). PEG was found to produce levels of formaldehyde that neared those seen by traditional combustion cigarettes, with one puff exposing the user to 1.12% of the daily exposure limit set forth by the United States Occupational and Safety Health Administration (OSHA) (Troutt and DiDonato, 2017). Squalene, a clear, slightly yellow oily substance, has been implicated in causing exogenous lipid pneumonia (Cha et al., 2019).

Recent studies have documented the presence of both VE and VEA in THC products (Taylor et al., 2019; Muthumalage et al., 2020a; Duffy et al., 2020). VEA is thought to be used as a diluent that mimics the consistency of a high purity THC oil, thus deceiving the consumer into believing they are purchasing a high-grade THC product and allowing a larger profit for the manufacturer by extending the supply (Duffy et al., 2020). VEA is also implicated as one of the primary chemicals thought to be responsible for the EVALI epidemic (Blount et al., 2019; Blount et al., 2020; Muthumalage et al., 2020b). Though VEA was not identified in any samples, VE was identified in multiple cannabinoid-containing products. Both VE and VEA produce quinone-like compounds, which can produce reactive oxygen species that increase cytotoxicity (Jiang et al., 2020).

### Flavorants

Trends in the use of chemicals used as flavorants are difficult to discern due to limited sample size (241 products) compared to products available commercially, the variety of chemicals available, the constantly changing formulations, and evolving product regulations. Though most flavorants have been designated as GRAS, the majority of the flavorants identified through screening are associated with at least one GHS classification, such as irritant, corrosive, or acutely toxic, which contraindicates their safety.

Several studies have demonstrated that flavorants produce varying degrees of toxicity to cells through different mechanisms (Behar et al., 2016; Leigh et al., 2016; Sherwood and Boitano, 2016; Behar et al., 2018). Omaiye et al. reported total concentrations of flavor chemicals in refill fluids were found as high as 362.3 mg/ml, while pod and cartomizer formulations evaluated (JUUL and Vuse, respectively) only contained total flavor chemical concentrations of 0.2–15.7 mg/ml (Omaiye et al., 2019a). They also reported that all 8 evaluated JUUL liquid formulations and the corresponding aerosols were cytotoxic. Cinnamaldehyde is already widely reported as cytotoxic, and a study evaluating cinnamaldehyde in e-liquids found that increasing battery output voltage further increased cytotoxicity (Behar et al., 2016). Costigan et al. pointed out that use of the same e-cigarette liquid in different devices will alter the aerosol formation (Costigan and Meredith, 2015). These studies’ findings suggest that a e-liquid toxicity may vary based on the device and operational settings.

Another flavorant-related health concern identified in this study is the formation of flavorant-carrier adduct products. Compounds such as vanillin propylene glycol acetal are formed post-production, sometimes within hours of initial product mixing, through an acetalization reaction between an aldehyde and PG. The formation of these acetal products was reported to be dependent on the ratio of PG present in the formulation (Erythropel et al., 2019). Adducts were shown to aerosolize with similar efficiency as the parent aldehyde and proved more effective at activating respiratory irritant receptors than the respective parent aldehyde, suggesting e-cig liquids may become more harmful to the user as time passes and flavorant-PG adducts form. The Flavor Extract Manufacturers Association (FEMA) lists some flavorant-carrier adducts as their own unique entity, which can be purchased for use as a flavorant (Flavor and Extracts Manufacturers Association of the United States, 2021). This can further complicate the determination of product ingredients in terms of what is added by the manufacturer or what was formed post-production. Additionally, if these chemicals are intentionally added, their concentration within the products may rise above the manufacturers intended level due to the post-production formation.

The combinations of flavorants may potentiate the harmful or toxic effects of one another. Manufacturers are currently not compelled to release e-liquid formulation recipes nor list all ingredients used within products on labels. Relevant toxicity studies are impossible to conduct as the proprietary formulations rapidly evolve.

### Nicotine

Nicotine, while a common constituent, is not always present. Some e-cig liquids are manufactured as nicotine-free, flavor-only options, and sometimes other pharmacologically active ingredients are used, as exemplified by the numerous cannabinoid liquid options available today. In this study, one e-liquid contained nicotine even though the product label claimed it did not contain any amount of nicotine. The presence of nicotine in this product could be due to contamination during the manufacturing process, incomplete/insufficient labeling, or the intention of including a known pharmacologically active ingredient to elucidate some effect. Other products were found to have higher or lower concentrations of nicotine than were labeled on the product, again demonstrating poor quality assurance and quality control standards in the industry (Peace et al., 2016; Peace et al., 2018).

Nicotine concentrations increased as manufacturers switched to nicotine salt formulations, which use nicotine with an organic acid, such as benzoic or lactic acid. These formulations allow manufacturers to significantly increase the nicotine concentration while reportedly reducing the harshness of such high nicotine amounts (Harvanko et al., 2020). The highest levels observed in this study were from JUUL liquid formulations, which use nicotine salts. The concentrations observed in these products correlate with concentrations identified in other studies (Omaiye et al., 2019a).

While nicotine use and dependence are well documented in the scientific literature, toxicity and poisonings, especially as it pertains to the e-cigarette industry, are worth discussing. Higher nicotine concentrations increase the chance of accidental nicotine poisoning, both through inhalation or ingestion of the liquid. The American Association of Poison Control Centers has reported thousands of poisoning cases about e-liquids since 2011, when e-cig use became more prevalent in the Unites States. As of May 31st of this year, 2063 cases have been reported (National Poison Data System, American Association of Poison Control Centers, 2021). Many poisoning cases involve young children ingesting the products accidentally, while some cases involve someone intentionally ingesting or injecting the liquid for a means of self-harm. Two cases of poisoning are reported following inhalation of a nicotine product by active duty military personnel, leading to clinical nicotine toxicity requiring emergency medical services (Bendel et al., 2021). Dermal contact from spilled or leaky pods is a concern that should not be overlooked, as nicotine readily absorbs through the skin, leading to both localized and systemic health concerns. Attempted homicide by nicotine liquid being poured directly onto the skin of the victim has been reported, with the victim describing the liquid as sticky with a spicy flavor (FOX 9, 2021).

### Caffeine

Caffeine was identified and quantitated in JUUL Menthol and Classic Tobacco liquid formulations. JUUL Classic Tobacco products contained an average of 23.5 μg/ml caffeine, while JUUL Menthol products contained an average of 9.3 μg/ml. In another study, caffeine in JUUL Menthol and Classic Tobacco aerosols were found in concentrations of 0.037 and 0.090 mM, respectively, showing it is able to both aerosolize and be inhaled by users (Omaiye et al., 2019a). Caffeine was not labeled on the e-liquid products.

Caffeine affects the cardiovascular, renal, nervous, and respiratory systems (Ueno et al., 2020), and is widely consumed throughout the world as a legal stimulant. In addition to the caffeine consumed in coffees, teas, sodas, and energy drinks, it can be found as a dietary supplement and is used in narcolepsy and asthma therapies.

Issues regarding caffeine in e-cig liquids should be considered. Inhalation of caffeine increases its bioavailability. An *in vivo* study using mice demonstrated caffeine inhalation, via a nebulizer, was an effective way of administering caffeine and produced greater spontaneous activity compared to the same dose administered intraperitoneally (Ueno et al., 2020). Additionally, caffeine was identified as an unlisted ingredient. Individuals with caffeine sensitivity or underlying medical conditions that require a caffeine-free lifestyle could be endangered by inhaling caffeine.

The addition of caffeine to e-cig liquids could act as an initiation primer, leading to increased caffeine seeking and consumption and chances of caffeine addiction. Caffeine consumption has been reported to increase the odds of smoking, the urge to smoke, and the subjective reinforcement from smoking (Treloar et al., 2014). A correlation between combined inhalation of caffeine and smoking with promotion of coronary heart disease and severe vascular lesions has also been reported (Pan et al., 2021).

### Menthol

Menthol was identified in a variety of products evaluated, sometimes as a listed ingredient, sometimes unlisted. Menthol was quantitated in concentrations as high as 4.48 mg/ml. Another study reported finding menthol in concentrations up to 68 mg/ml (Omaiye et al., 2019b). In that study, the cytotoxic properties of several flavorants were evaluated. Menthol was identified as toxic in cells studied in concentrations 30 times lower than their highest identified concentration, meaning all products evaluated with menthol in concentrations greater than approximately 2.5 mg/ml would be considered cytotoxic.

Menthol has been identified as having other important pharmacological properties related to smoking and vaping. Therefore, its identification as an additive in non-menthol flavored products is not surprising. Menthol is reported as imparting a cooling sensation with analgesic or counterirritant effects, reducing the perceived harshness of the nicotine and smoke or aerosol (Ton et al., 2015; Taylor et al., 2021). Menthol’s effects are thought to allow users to inhale deeper, hold the smoke or aerosol in the lungs longer, and use products with higher nicotine content, all which may allow toxic or carcinogenic chemicals to have a longer duration of exposure (Ton et al., 2015). Menthol, while itself is an irritant, can also work as a counterirritant for other chemicals commonly encountered in e-cig liquids.

Menthol has been reported to reduce nicotine metabolism both *in-vitro* and *in-vivo,* leading to increased systemic nicotine exposure, reduced clearance, and longer durations of pharmacological effects (Benowitz et al., 2004; Alsharari et al., 2015)*.* Menthol has been associated with reduced Cytochrome-P450 2A6 isoform activity (MacDougall et al., 2003; Benowitz et al., 2004). This same enzyme is responsible for nicotine metabolism, thus co-ingestion of menthol with nicotine will alter nicotine metabolism and elimination and therefore prolong the pharmacological effect of nicotine felt by the consumer.

### Ethanol

Ethanol has been identified in many of the products evaluated in this study, including nicotine refill formulations, nicotine pods, and DOTN formulations, with concentrations ranging from not-present up to 217.2 mg/ml. Previous studies have also reported on ethanol concentrations in e-cig liquids, and have also identified concentrations of ethanol greater than 20% (Valentine et al., 2016; Poklis et al., 2017b).

Some flavoring chemicals use ethanol as part of the manufacturing process, such as vanilla, which is required by FDA Code of Federal Regulations to contain “not less than 35% by volume” (United States Food and Drug Administration, 2020a). While ethanol may be present in formulations due to their use in flavorings, it can also be added to thin the liquid, to help dissolve other substances that are not miscible with typical carriers, or for intentional consumption of ethanol. Do-it-yourself (DIY) e-liquids may contain higher concentrations of ethanol than those found in the manufactured products reported in the literature.

There is a dearth of knowledge regarding ethanol pharmacokinetics and intoxication from inhalation. Inhalation bypasses first-pass metabolism and increases bioavailability of ethanol. It has also been suggested that co-administration of ethanol with nicotine could lead to increased dependence and addiction liability for both substances due to their synergistic nature (McKee et al., 2006; Oliver et al., 2013), especially as ethanol has been found to potentiate several of the rewarding effects of nicotine (Rose et al., 2004).

A clinical study from 2017 compared the effects of inhaling a nicotine e-liquid with high or trace amounts of alcohol (Valentine et al., 2016). They found no difference in subjective effects between high and trace alcohol groups. Their findings suggest that users of high alcohol concentration e-cig liquids may experience some alteration in psychomotor function without recognizing any subjective effects to alert them to the impairment. Additionally, they found the metabolites of ethanol in the urine of three participants out of the eight exposed to the high alcohol e-liquid.

### Olivetol

Olivetol was identified and quantitated in five cannabinoid-based e-liquid products. Concentrations ranged from below the limit of quantitation to 3.9 mg/ml. Olivetol is a naturally occurring organic compound that can be used as a precursor for synthesizing various cannabinoids (Tadayon and Ramazani, 2021). Commonly found in lichen, it also exists for a short time in cannabis plants before conversion to CBGA, the precursor to THCA and CBDA. Its presence in e-liquid products may be due to an incomplete chemical reaction used by manufacturers when synthesizing THC, and thus could be considered an impurity. Olivetol may also be an intentional ingredient in e-liquid products. It is touted as an “antidote” to purportedly reduce unwanted effects from the consumption of high concentration THC products, such as anxiety, paranoia, or feeling overly “high” from a THC overload (Carberry, 2018; Royal Queen Seeds, 2019). Olivetol is thought to act on cannabinoid CB1 and/or CB2 receptors (Carberry, 2018). Evidence of olivetol’s ability to reduce some effects of THC was reported in the UNDOO, LLC product patent application (Carberry, 2018). No clinical trials evaluating olivetol’s effects have been conducted to support these claims. UNDOO reports volunteer testimonies from real-life, non-clinical trials in which participants either smoked or ingested THC, then took known amounts of olivetol, and reported on their subjective experiences.

Olivetol is listed in the National Institute of Health’s PubChem database as a GHS irritant, causing skin, eye, mucous membrane, and respiratory irritation that could lead to severe tissue destruction and specific target organ toxicity with a single exposure ( National Institute of Health, 2021 ). It emits carbon monoxide and carbon dioxide when heated to decomposition, and it is recommended to use a NIOSH-approved respirator equipped with an organic vapor/acid gas cartridge when handling neat olivetol (National Oceanic and Atmospheric Administration, 2021).

### Cannabinoids

Cannabinoids have been identified and quantitated in several products evaluated in this study. CBD and THC were the most abundant in both prevalence and concentration and were quantitated in concentrations as high as 332.6 and 497.7 mg/ml, respectively. Cannabinoid-based e-liquid formulations have existed since the advent of the modern e-cigarette. JUUL is a spin-off company and product of PAX, a discreet cannabis vaporizer launched in 2012. Grenco Science also officially launched the THC vaporizer in 2012, after years of product development and testing (Freedman, 2014; Farah, 2017; Bobrow, 2019; Hartman, 2021). The launch of these cannabis-based e-cigs coincided with adult-use legalization in Colorado and Washington. Even though almost every state in the United States has legalized some form of C. sativa, whether medical or adult use, the regulation of cannabis and cannabis products vary by the state.

The National Institute on Drug Abuse (NIDA) Monitoring the Future survey data indicates that while “any vaping”, “vaping nicotine”, and “vaping flavors” trends appear to be steady, or maybe even slightly decreasing, between 2019 and 2020, “vaping marijuana” is still on the rise among 8th, 10th, and 12th graders (National Institute of Health, 2020).

Like other chemicals, cannabinoid labeling accuracy has significant deficiencies. In this study, products were analyzed that were labeled with the wrong concentration, or listed as THC-free, though THC was identified. Supplementary Material D lists bottle/packaging claims and results of screening and cannabinoid quantitation. Eight out of nine products analyzed that indicated total volume of product or cannabinoid content were over-labeled. By comparison, one study reported nearly half of the CBD products analyzed were under-labeled and about 25% were over-labeled, reporting “vaporizing liquids” to be the most frequently mislabeled (85%) and oil the most frequently labeled accurately (Bonn-Miller et al., 2017). Additionally, they reported THC was present in 18 of 84 products tested, though they were listed as THC free. The current study found THC in five samples out of nine that indicated they were “THC free” or contained “0% THC”. Inaccurate product labeling demonstrates the lack of quality assurance and quality control required in the industry and poses a significant danger to consumers who may consume a higher dose than intended.

Chronic use of products containing trace concentrations of THC can result in failed urine drug tests (Spindle et al., 2020; Sholler et al., 2021). Additionally, higher peak blood concentrations have been reported from vaping THC compared to the same dose of smoked cannabis (Spindle et al., 2019). This increase in delivery efficiency is thought to be a product of minimized sidestream smoke and lack of drug pyrolysis, both of which reduce the possible dose to be inhaled in traditional combustion delivery methods (Pomahacova et al., 2009). Compared to combustion smoking conditions, vaping cannabis has been found to increase the frequency of testing above immunoassay cutoff levels in a clinical setting (Spindle et al., 2020).

Some marketed “cannabis” products contain synthetic novel psychoactive substances (NPS) as the active drug. Many NPSs are not scheduled, and therefore legal, at the time of product manufacturing. By the time these chemicals are identified and federally scheduled, manufacturers have adopted another NPS which is not scheduled, allowing manufacturers to skirt federal DEA regulations. These NPS can be more potent and can lead to severe and life-threatening situations. Some consumers have information that certain products contain NPSs and knowingly choose to use those products. Some consumers are unaware that products purchased for relief contain NPSs but experience untoward effects (Poklis et al., 2019).

Cannabinoid-based products evaluated in this study have generally become more complicated over the years. Early formulations were mainly comprised of PG, VG, cannabinoids, and terpenes. More recent formulations contain a variety of carriers, sometimes mixing multiple carriers in one product, as well as extra active ingredients and flavorants. Terpenes found in older products are thought to be carried over from extraction methods (Peace et al., 2016). Recent formulations market terpene-specific profiles to appeal to flavor preferences or purported health benefits. Until these products are federally regulated, formulations can only be limited to individual States’ regulations.

### Lung Injury

The carriers, diluents, thickeners, flavorants, and solvents identified have been generally considered as safe because of their accepted safety for oral ingestion, yet there is minimal to no evidence for long-term consequences from inhaling these substances. These chemicals can create injury to the lung tissue. They can prevent proper oxygen flow, disrupt cell membranes, cause irritation and inflammation to the lung tissue, mucosa, and bronchi, and induce lipoid pneumonia ( Erythropel et al., 2019; Thirion-Romero et al., 2019; Muthumalage et al., 2020b ). Two mechanisms for lung pathogenesis have been proposed. The first hypothesis describes acute exposure that creates a direct chemical injury that results in negative health effects (Alexander et al., 2020). The second mechanism describes a change to the immune cells in the alveoli due to chronic exposure to a chemical, which may or may not result in symptomology recognized by the vaper. With the addition of a new chemical, the body reaches some threshold that triggers a pathologic inflammatory response, precipitating neutrophil recruitment, edema, and necrosis (Johnson and Matthay, 2010; Alexander et al., 2020).

## Conclusion

Increasing concentrations of pharmacologically active ingredients, the risk for complex drug-drug interactions both from individual products and co-administration with other drugs, and the general unknown implications of vaping GRAS chemicals underscore the need for transparent reporting of chemical constituents. The absence of regulatory oversight of specific ingredients and labeling requirements make the demonstration of general safety of such products difficult. Unsuspecting consumers can and are experiencing untoward and unexpected effects. Physicians may not understand and attribute the etiology of reported symptoms, leading to misdiagnoses and/or incomplete treatment regimens. Continued studies evaluating chronic and acute exposure of both singular ingredients and chemical mixtures are critical. With these considerations, constant product surveillance incorporating untargeted chemical analyses of products intended for public consumption is critical for understanding what chemical ingredients are being used in these products and the potential health and safety impacts.

## Data Availability

The original contributions presented in the study are included in the article/Supplementary Material. Further inquiries can be directed to the corresponding author.
