# Supplementary material for: A Retrospective Analysis of Chemical Constituents in Regulated and Unregulated E-Cigarette Liquids
Source: Front Chem. 2021 Oct 28;9:752342. doi: 10.3389/fchem.2021.752342 (PMC8581558; doi:10.3389/fchem.2021.752342)
Supplement: Supplementary file 1 [file DataSheet1.docx]

**SUPPLEMENTARY MATERIAL A: Methods**

**Screening by DART-MS**

Screening of e-liquids was performed on a JEOL JMS T100LC Accu-TOF DART-MS operated in positive-ion mode and controlled by Mass Center software version 1.3.4 m (JEOL Inc., Tokyo, Japan) following previously published parameters (Poklis et al., 2015). In brief, a capillary tube was dipped directly into the e-liquid and then introduced into the helium stream. The helium stream temperature was set to 300°C with a flow rate of 2.0 L/min, a discharge electrode needle voltage of 4000 V, and the grid electrode was set to 250 V. The ion guide peak voltage was 400 V, reflectron voltage was 900 V, orifice 2 was set to 5 V, and the ring lens was set to 3 V with orifice 1 operating in function switching mode at 20, 30, 60, or 90 V. The masses measured ranged from 40 to 1100 Da. The data was analyzed by creating an averaged, background subtracted, centroided mass spectra that was calibrated using PEG 600. Data was evaluated using National Institute of Standards and Technology (NIST) and Scientific Working Group for the Analysis of Seized Drugs (SWGDRUG) libraries loaded into Mass Mountaineer software (Diablo Analytical, Inc., Antioch, CA, USA). Presumptive identifications were made when the exact mass was detected within 5 mDa of a compound’s calculated monoisotopic mass (M+H)^+^.

**Screening by GC-MS**

E-liquids were screened by making a 1:100 dilution in methanol and analyzing using an untargeted GC-MS base screen method. Screening was performed on a Shimadzu QP-2020 GC-MS (Kyoto, Japan) controlled by GCMS Real Time Analysis version 4 (Shimadzu Corp. Kyoto, Japan). The chromatographic separation was performed on a HP-5MS column (30 m × 0.25 mm id × 0.25 μm) (Agilent, Santa Clara, CA). The GC-MS was operated in splitless mode using 1 μL sample injections. The carrier gas was helium at a linear velocity of 35 cm/s. The oven temperature was programmed to hold for 1 minute at 70°C, then ramped at 15°C per minute to 300°C and held for 10 minutes for a total run time of 26.33 minutes. The scan range for the mass spectrometer was 40–550 *m/z*. A test mix containing 10 compounds was injected prior to samples. Correct retention time, peak shape, and abundance were evaluated to ensure the instrument was operating optimally. Methanol blanks were injected between samples to check for contamination or carryover. NIST and SWGDRUG libraries were used for identification of compounds, which could be compared to DART-MS results.

**Quantitation of Nicotine by GC-MS**

Calibrators were prepared at 10, 20, 50, 100, 200, and 500 µg/mL nicotine in methanol. The limit of quantitation (LOQ) was defined as the lowest calibrator, which produced a signal-to-noise ratio greater than 10 and had a concentration of 10 μg/mL with a linear range of 10-500 µg/mL. Lots of matrix matched quality controls (QCs) were prepared at 30, 150, and 300 µg/mL nicotine by adding 5 µL of a 30:70 PG:VG mixture as part of the total 100 µL volume. E-cigarette liquids were diluted 1:100 in methanol. Samples that came in above the calibration curve were further diluted in methanol until they fell withing the working calibration range. Calibrators, controls in triplicate, blank matrix with and without internal standard and samples were prepared for analysis by adding 100 µL to a vial with a glass insert with 15 µL internal standard (200 µg/mL quinoline in methanol).

Quantitation of nicotine was accomplished by GC-MS using a Shimadzu QP-2020 GC-MS (Kyoto, Japan) controlled by GCMS Real Time Analysis version 4 (Shimadzu Corp. Kyoto, Japan) following previously published parameters (Pagano et al., 2016). The chromatographic separation was performed on a HP-5MS column (30 m × 0.25 mm id × 0.25 μm) (Agilent, Santa Clara, CA). Helium carrier gas was run through a split injector (50:1 split) at a temperature of 230°C. The injection volume was 1 µL of sample. The initial oven temperature was 80°C held for 1 min and then ramped at 25°C per minute to 245°C and held for 4.5 minutes for a total run time of 11.10 minutes. The source and transfer line were kept at 180°C and 280°C, respectively. Single-ion-monitoring (SIM) mode was used, monitoring *m/z* values of 84 (quantitation), 133, and 162 for nicotine and 129 (quantitation) and 102 for quinolone. Methanol blanks were evaluated for contamination and carryover. Imprecision, accuracy, and measurement of uncertainty were determined by evaluating low (30 μg /mL), mid (150 μg /mL) and high (300 μg/mL) QC concentrations. Each was repeated three times on three different days for intraday precision. Accuracy was calculated as the percent of the target concentration and imprecision was calculated as the percent coefficient of variation (%CV). Accuracy and %CV were both within 15%. Instrument repeatability was calculated by injecting each QC 3 times and calculating the mean and standard deviation. Bias was calculated as the percent difference between the calculated values and actual values and was within ±15%. Carryover was assessed by injecting a double blank after the highest calibrator and between QC samples and product samples. Carryover was not detected. Matrix effects were not observed.

**Quantitation of Caffeine and Menthol by GC-MS**

Mixed caffeine and menthol calibrators were prepared at 100, 200, 500, 1000, 2000, and 5000 ng/mL in methanol. The limit of quantitation (LOQ) was 100 ng/mL with a linear range of 100-5000 ng/mL. Mixed QCs were prepared at 300, 800, and 4000 ng/mL caffeine and menthol. E-cigarette liquids were serial diluted 1:10, 1:100, 1:1000, and 1:10,000 in methanol to ensure target analytes fell within the working curve range. Calibrators, controls in triplicate, blank matrix with and without internal standard and samples were prepared for analysis by adding 100 µL to a vial with a glass insert with 10 µL mixed internal standard (10 µg/mL caffeine-(trimethyl-d9) and trans-anethole in methanol).

Quantitation of caffeine and menthol was accomplished by GC-MS using a Shimadzu QP-2020 GC-MS (Kyoto, Japan) controlled by GCMS Real Time Analysis version 4 (Shimadzu Corp. Kyoto, Japan). The chromatographic separation was performed on a HP-5MS column (30 m × 0.25 mm id × 0.25 μm) (Agilent, Santa Clara, CA). Helium carrier gas was run through a split injector (20:1 split) at a temperature of 250°C. The injection volume was 1 µL of sample. The initial oven temperature was 70°C held for 1 min, then ramped at 15°C per minute to 220°C followed by ramping at 30°C per minute to 300°C for a total run time of 13.67 minutes. The source and transfer line were kept at 250°C and 280°C, respectively. Single-ion-monitoring (SIM) mode was used, monitoring *m/z* values of 71 (quantitation), 81, 95, and 123 for menthol, 148 (quantitation), 105, 117, and 147 for trans-anethole, 203 (quantitation), 60, 70, and 115 for caffeine-(trimethyl-d9), and 194 (quantitation), 55, 67, and 109 for caffeine. Methanol blanks were evaluated for contamination and carryover. Imprecision, accuracy, and measurement of uncertainty were determined by evaluating low (300 ng/mL), mid (800 ng/mL) and high (4000 ng/mL) QC concentrations. Each was repeated three times on three different days for intraday precision. Accuracy was calculated as the percent of the target concentration and imprecision was calculated as the % CV. Accuracy was within 20% and %CV was within 20%. Instrument repeatability was calculated by injecting each QC 3 times and calculating the mean and standard deviation. Bias was calculated as the percent difference between the calculated values and actual values and was within ±20%. Carryover was assessed by injecting a double blank after the highest calibrator and between QC samples and product samples. Carryover was not detected.

**Quantitation of Vitamin E and Vitamin E Acetate by GC-MS**

Mixed Vitamin E and VEA calibrators were prepared at 10, 20, 50, 100, 200, 500, 1000, and 2000 ng/mL in methanol. The limit of quantitation (LOQ) was 10 ng/mL with a linear range of 10-2000 ng/mL. Mixed QCs were prepared at 30, 300, and 750 ng/mL Vitamin E and VEA. Samples were serial diluted in methanol to ensure target analytes fell within the working curve range. Calibrators, controls in triplicate, blank matrix with and without internal standard and samples were prepared for analysis by adding 100 µL to a vial with a glass insert with 10 µL internal standard (10 µg/mL Vitamin E-d6 in methanol).

Quantitation of Vitamin E and VEA was accomplished by GC-MS using a Shimadzu QP-2020 GC-MS (Kyoto, Japan) controlled by GCMS Real Time Analysis version 4 (Shimadzu Corp. Kyoto, Japan). The chromatographic separation was performed on a HP-5MS column (30 m × 0.25 mm id × 0.25 μm) (Agilent, Santa Clara, CA). Helium carrier gas was run through a splitless injector at a temperature of 250°C. The injection volume was 1 µL of sample. The initial oven temperature was 200°C followed by ramping 50°C per minute to 300°C with a four-minute hold for a total run time of 6.00 minutes. The source and transfer line were kept at 250°C and 280°C, respectively. Single-ion-monitoring (SIM) mode was used, monitoring *m/z* values of 165 (quantitation), 430, and 205 for Vitamin E, 165 (quantitation), 430, and 205 for VEA, and 171 (quantitation), 436, and 211 for Vitamin E-d6. Methanol blanks were evaluated for contamination and carryover. Imprecision, accuracy, and measurement of uncertainty were determined by evaluating low (30 ng/mL), mid (300 ng/mL) and high (750 ng/mL) QC concentrations. Each was repeated three times on three different days for intraday precision. Accuracy was calculated as the percent of the target concentration and imprecision was calculated as the % CV. Accuracy was within 20% and %CV was within 20%. Instrument repeatability was calculated by injecting each QC 3 times and calculating the mean and standard deviation. Bias was calculated as the percent difference between the calculated values and actual values and was within ±20%. Carryover was assessed by injecting a double blank after the highest calibrator and between QC samples and product samples. Carryover was not detected.

**Quantitation of Volatiles by HS-GC-FID**

E-cigarette liquids were diluted 1:10 in deionized (DI) water. Mixed calibrators were prepared at 100, 300, 500, 900, 1500, and 3000 µg/mL of acetone, ethanol, isopropanol, and methanol in water. The limit of detection (LOQ) for all volatiles was 100 mg/L with a linear range of 100-3,000 mg/L. Lots of matrix matched mixed QCs were prepared at 200, 600, and 2000 µg/mL acetone, ethanol, isopropanol, and methanol in a 30:70 PG:VG mixture. Calibrators, controls in triplicate, blank matrix with and without internal standard and samples were prepared for analysis by adding 100 µL to a headspace vial with 1 mL internal standard (20 mg/L t-butanol in water).

Quantitation of acetone, ethanol, isopropanol, and methanol was accomplished using a modified previously published method for headspace gas chromatography-flame ionization detector (HS-GC-FID) and employed a Shimadzu HS-20 headspace sampler attached to a Nexis 2030 GC-dual FID controlled by LabSolutions software (Shimadzu Corp., Kyoto, Japan) (Poklis et al., 2017b). The chromatographic separation was performed on RTX-BAC PLUS 1 (30 m× 0.32 mm id × 1.80 µm) and RTX-BAC PLUS 2 (30 m× 0.32 mm id × 0.6 µm) columns (Restek Corp, Bellefonte, PA, USA). The sample line and transfer line temperatures were set to 170°C, and the platen temperature was 80°C with the mixer on. The incubation time was 5 min with a sample injection time of 0.5 min. The GC oven temperature was set to 40°C with an injection temperature of 200°C run in split mode with a 1:20 ratio. The column ﬂow rate was 2.57 mL/min with purge ﬂow of 0.5 mL/min, and the detector temperature was 225°C. Analyte values were averaged between the two FIDs. Accuracy was within 15% and %CV was within 15%. Instrument repeatability was calculated by injecting each QC 3 times and calculating the mean and standard deviation. Bias was calculated as the percent difference between the calculated values and actual values and was within ±15%. Carryover was assessed by injecting a double blank after the highest calibrator and between QC samples and product samples. Carryover was not detected.

**Quantitation of Olivetol by LC-MS/MS**

Due to the viscous nature of some products, most products were diluted for analysis by transferring a small amount of sample into a test tube, collecting the mass, and adding a small volume of methanol. Test tubes were capped and vortexed vigorously to dissolve and homogenize the sample. Calibrators were prepared at 100, 200, 500, 1000, 2000, and 5000 ng/mL in methanol. QCs were prepared at 300, 800, and 4000 ng/mL in methanol. Calibrators, controls in triplicate, blank matrix with and without internal standard and samples were prepared for analysis by adding 100 µL to a vial with 10 µL internal standard (10 µg/mL olivetol-d9 in methanol). The limit of quantitation (LOQ) for olivetol was 100 ng/mL with a linear range of 100 to 5000 ng/mL.

Quantitation of olivetol was accomplished using a Shimadzu LC-MS 8050 controlled with LabSolutions software (Shimadzu Corp., Kyoto, Japan). Chromatographic separation was performed on a Zorbax Eclipse XDBC18 column 4.6 × 75 mm, 3.5 micron (Agilent Technologies, Santa Clara, CA) held at 40°C. Five mM ammonium formate in water with 0.1% formic acid (A) and methanol (B) were used as the mobile phase. A binary gradient was used with a flow rate of 1.0 mL/min and the following programming: 0.00-0.01 min: 5% B; 0.02-9.00 min: 95% B; 9.01-9.50 min: 95% B; 9.51-10.50 min: 5% B. The source temperature was set at 650°C and had a curtain gas flow rate of 30 mL/min. The ion spray voltage was 5,000 V, with the ion source gases 1 and 2 at flow rates of 60 mL/min. The following transition ions (*m/z*) were monitored in multiple reaction monitoring (MRM) mode (Lens 1 Voltage, Collision Energy, Lens 3 Voltage): olivetol, 181.0>111.0 (-27.0, -14.0, -20.0) and 181.0>71.0 (-13.0, -13.0, -11.0); olivetol-d9, 190.0>112.0 (-30.0, -14.0, -18.0) and 190.0>80.0 (-15.0, -15.0, -12.0).

**Quantitation of Cannabinoids by LC-MS/MS**

E-cigarette liquids were serial diluted in methanol to ensure analytes fell within the working calibration range. Mixed calibrators of the 9 cannabinoid analytes (CBD, Δ9-THC, CBG, CBC, CBN, THCA, Δ8-THC, CBDA, and THCV) were prepared at 10, 20, 50, 100, 200, 500, and 1000 ng/mL in methanol. Mixed QCs were prepared at 30, 300, and 750 ng/mL in methanol. Calibrators, controls, blank matrix with and without internal standard and samples were prepared for analysis by adding 100 µL to a vial with 10 µL mixed internal standard (1 µg/mL CBD-d3, Δ9-THC-d3, and CBN-d3 in methanol). The limit of detection (LOQ) for all cannabinoid analytes was 10 ng/mL with a linear range of 10-1000 ng/mL.

Quantitation of cannabinoids was accomplished using a Shimadzu LC-MS 8050 controlled with LabSolutions software (Shimadzu Corp., Kyoto, Japan) using a modified version of a previously published method (Poklis et al., 2010). Chromatographic separation was performed on a Zorbax Eclipse XDBC18 column (4.6 × 75 mm, 3.5 micron, Agilent Technologies, Santa Clara, CA) held at 40°C. An isocratic method was used with a flow of 0.15 mL/min for mobile phase A (10mM ammonium formate in water with 0.1% formic acid) and 0.85 mL/min for mobile phase B (methanol). The source temperature was set at 650°C and had a curtain gas flow rate of 30 mL/min. The ion spray voltage was 5,000 V, with the ion source gases 1 and 2 at flow rates of 60 mL/min. Transition ions (*m/z*) monitored in multiple reaction monitoring (MRM) mode can be found in Appendix B. The chromatographic method resolved analytes with identical transition ions.
